# Contraceptive preferences and unmet need for contraception in midlife women: where are the data?

**DOI:** 10.1186/s40695-017-0026-6

**Published:** 2017-09-11

**Authors:** Siobán D. Harlow, Jennifer R. Dusendang, Michelle M. Hood, Nancy Fugate Woods

**Affiliations:** 1 Department of Epidemiology, University of Michigan, Ann Arbor, Michigan USA; 2 Biobehavioral Nursing and Health Informatics, University of Washington, Seattle, Washington USA; 3 Department of Epidemiology, University of Michigan 1415 Washington Heights, Suite 6610 SPH I, Ann Arbor, Michigan USA

**Keywords:** Contraception, Menopause, Midlife, Women’s health

## Abstract

This commentary discusses the limited availability of information on contraceptive preferences and unmet need for contraception among midlife women in both high and low income countries. Given that risk of pregnancy continues until women reach menopause and given the increased risk of pregnancy complications, elective abortion, and maternal mortality in women aged 45 to 54 years old, increased focus on gathering basic data on midlife women’s preferences and unmet need is warranted.

Midlife marks a period of declining fertility as women approach and complete the transition to menopause [1]. With reproductive aging, the number of antral follicles decline and the ovary becomes less responsive to follicle stimulating hormone (FSH), leading to increasing irregularity in menstrual periodicity, more frequent luteal phase insufficiency and anovulation. Nonetheless, the possibility of pregnancy continues until women reach menopause, as relatively normal ovulatory cycles have been documented to occur up until the time of the final menstrual period (FMP) [2, 3]. It is recommended that women aged 50 to 55 years of age continue contraception until after at least 1 year of amenorrhea and that women under 50 years continue until after at least 2 years of amenorrhea [4, 5]. Although much attention has been paid in recent years to enhancing the fertility potential of women in their 40s who are seeking to become pregnant [6], few studies have addressed the unmet need for contraception or evaluated the contraceptive preferences of midlife women. Alkema and colleagues [7] recently published a comprehensive global review of the prevalence of contraceptive use and unmet need by country using the United Nations Population Division database, however, that report did not disaggregate the data by age.

## US patterns

Data are particularly limited for midlife women in high-income countries. For example, the median age at menopause in the United States (US) is 51.4 years [8], thus approximately 50% of women in their early to mid-50s, who have not had a hysterectomy, remain at potential risk of pregnancy. Yet, few studies in the US are able to provide information on contraceptive practices of women older than 45 years of age. The National Survey of Family Growth has historically collected data on women aged 15 to 44, although in 2015 the eligible age range was expanded to 15–49 years [9]. The Behavioral Risk Factor Surveillance System (BRFSS) does not administer the family planning module to women aged 45 years and older, with the exception of the state of Massachusetts [9]. Large cohort studies, such as the Nurses’ Health Study and the Study of Women’s Health Across the Nation (SWAN), obtained information only on hormonal forms of contraception as their interest was in understanding health risks of hormone exposures and hormone therapy practices for symptoms of menopause. The National Health and Nutrition Examination Survey (NHANES) provides data for women up through age 54 years, however, this survey inquired only about hormonal forms of contraceptive use, specifically oral contraceptive pills and depo-provera [10].

In the 2006–2008 National Survey of Family Growth, 78% of women aged 40–44 reported using contraception, with sterilization the most frequently reported among those using contraception (50.2% female and 19.6% male) [11]. Oral contraceptive use was reported by 11.1% with only 1.1% reporting use of injectable contraception. Condom use was reported by 8.8%, the intrauterine device by 4.2%, and other methods including natural family planning by 5.1%. It was estimated that 7.6% of women in this age group are at risk of pregnancy and not using contraception [11].

The Massachusetts BRFSS ascertained contraceptive use for women aged 18 to 50 years old [9]. Although women aged 45–50 were less likely to be sexually active and more likely to have had a hysterectomy than younger women, 78% remained potentially at risk of unintended pregnancy. Of these potentially at-risk women, 67% reported using some form of contraception: 44% had had a tubal ligation or their partner had had a vasectomy, 3.3% used intrauterine devices or implants, 5.9% used hormonal contraceptives, 11.6% used barrier methods and 2.1% used other methods. At-risk women in this age group were more likely not to use contraception than younger women (16.8% as compared to 9.3% of 18–24 year olds, 11.3–11.7% of 25 to 39 year olds, and 14.7% of 40–44 year olds). These data are specific to the state of Massachusetts and may not reflect the national experience: The reported frequency of hysterectomy (9.2%) is lower than the national average. Access to contraceptive services also differs state by state.

The NHANES data are limited to information on use of birth control pills and the injectable depo-provera [10]. We used data from the 2011–2012 survey [12] to assess potential unmet need for contraception and use of these two types of contraceptives among women aged 40 to 54 years of age. Table 1 provides information on the percent of women who are at risk of pregnancy (women who are sexually active, have not had a hysterectomy and are not postmenopausal), with women aged 35–39 included for comparison. The percent of women at risk of pregnancy declines from 70% of women aged 40–44 to 56% of women aged 45–49 to 24% of women aged 50–54 years old. Among at risk, sexually active women, oral contraceptive pill (OCP) use was reported more frequently than depo-provera (See Table 2). OCP use was reported by 13% of the at-risk 40 to 44 year olds and 17.6% of the at-risk 45–49 year olds compared to less than 1 % for depo-provera. Less than 2% of the at-risk 50–54 year olds reported use of either method. Depo-provera use was more frequent in Black and Hispanic women (prevalence <3%) than in White women. Notably, most at-risk women aged 40–54 years did not use either method. Although somewhat informative about national contraceptive practices, these data are limited by the lack of information on permanent sterilization, barrier methods and other forms of contraception. Also, the weighted data are based on just 743 women, just 427 of whom were sexually active and at risk of pregnancy, making it difficult to conduct further subgroup analysis.

**Table 1:** Weighted percent of US women aged 35 to 54 years who are at risk of pregnancy and sexually active, the National Health and Nutrition Examination Survey, 2011–2012 [10]^a^

Age	Have had a hysterectomy	Postmenopausal	Not sexually active	At risk^b^
35–39	7.3%	0.0%	7.4%	85.4%
40–44	21.8%	0.2%	8.2%	69.8%
45–49	24.0%	7.7%	12.4%	55.9%
50–54	29.5%	35.8%	10.6%	24.1%

^a^National Health and Nutrition Examination Survey, 2011–2012 https://wwwn.cdc.gov/nchs/nhanes/ContinuousNhanes/Default.aspx?BeginYear=2011 [10]

^b^At-risk women include women who are sexually active, have not had a hysterectomy and are not postmenopausal

**Table 2:** Weighted percent of US women aged 35 to 54 years at risk of pregnancy and sexually active who are using oral contraceptive pills or depo-provera, the National Health and Nutrition Examination Survey, 2011-2012^a^

Age	Oral contraceptive pill	Depo-provera	Both	Neither
35–39	11.3%	0.8%	0.9%	86.9%
40–44	13.0%	0.7%	0.0%	86.3%
45–49	17.6%	0.0%	0.0%	82.4%
50–54	0.8%	0.9%	0.0%	98.3%

^a^National Health and Nutrition Examination Survey, 2011–2012 https://wwwn.cdc.gov/nchs/nhanes/ContinuousNhanes/Default.aspx?BeginYear=2011 [10]

These limited US data, along with data on elective abortions [13], suggest that an appreciable percentage of midlife women potentially have an unmet need for contraception. This unmet need is particularly worrisome given the increased risk of pregnancy complications and maternal mortality in women age 40 and older [4]. In 2013, US birth rates were 10.4 births per 1000 women aged 40–44, 0.8 births per 1000 women aged 45–49, and 0.7 births per 10,000 women aged 50–54 [14]. However, these calculations do not take into account the fact that many women aged 40 and older are not at risk of pregnancy because of hysterectomy or menopause. Based on the NHANES data in Table 1, at most 68% of women aged 45–49 and 35% of women aged 50–54 are at risk of pregnancy, thus birth rates for these two age groups are likely closer to 1.2/1000 and 2/10,000 women at-risk, respectively.

Although only 15% of births are to mothers age 35 and older, over 27% of maternal death occur to mothers in this age group [15]. Significant race/ethnic disparities exist, with non-Hispanic- African American women having more than a threefold higher risk of a pregnancy related death than non-Hispanic whites (38.9 versus 12.0 deaths/100,000 live births respectively). This disparity increases with age reaching approximately 148 deaths per 100,000 live births for African American women aged 40 and older compared to approximately 35 per 100,000 for whites. Thus, information on desire for pregnancy, contraceptive preferences and barriers to access is of particular importance to address these health disparities.

## Global patterns

Information on contraceptive practices in other high-income countries is also limited. Most studies from Europe are 15–20 years old, have data only through age 44, and were based on small samples [4]. In 2003, a survey of over 12,000 women aged 15 to 49 years was conducted in France, Germany, Italy, Spain, and the United Kingdom but the report provides only limited information about age specific practices [16]. Sterilization was most common in women over age 40, while use of long-term, reversible contraception was most common in women aged 35–44. About one-third of women aged 45–49 did not use any form of contraception.

A survey on contraception and sexual health was undertaken by the United Kingdom’s Office for National Statistics in 2008/09 and included women aged 40–49 years old [17]. Most women used some contraception with 75% of 40–44 year olds and 72% of 45–49 year olds currently using contraception. Sterilization was the most commonly used form of contraception with 46 and 49%, respectively reporting that either they or their partner had been sterilized. Male partners were more likely to be sterilized than the women themselves. The next most commonly reported contraceptives were condoms (21 and 11%) and oral contraceptives (10 and 13%), respectively. Natural family planning/rhythm/withdrawal was reported by 10 and 9%, and the intrauterine device by 9 and 11%. Other types of hormonal contraceptives and other barrier methods were each used by 5% in both age groups.

A Canadian internet based survey of women aged 15 to 50 years who were sexually active and not pregnant [18] reported that among women aged 40 and older, 60% never used contraception while 33% always used contraception, with the 1.7% of women who had had a hysterectomy included in the always used category. Information on menopausal status was not available. Non-use of contraception was associated with lower income, lower education, rural residence and being married. Women were able to indicate use of more than one type of contraception. The most commonly used contraceptives reported by women of this age group were condoms (42.5%) and sterilization of either partner (36.0%). Oral contraceptive use was reported by 17.1%, while other types of hormonal contraceptives were used by 4.8%. Natural family planning/withdrawal was reported by 17.1% and barrier methods by 7.5%.

Considerably more data are available from low-income countries (LICs) given the historic focus of the Demographic and Health Surveys (DHS) on contraceptive behavior, although these surveys generally only include information on women up through age 49. The problem of unmet need in LICs has received considerable attention [5], but few papers specifically address the unmet need for midlife women [19]. Table 3 compiles data from the country-specific DHS survey reports from African countries surveyed between 2006 and 2015. The table provides information on the percent of women who report not using any contraception as well as fertility rates and, where reported, the estimated maternal mortality per 1000 women [20]. We focused on unmet need, given that contraceptive use remains low in most countries. In most countries, the percent of women not using contraceptives increases from age 40–44 to age 45–49 years, although fertility at these ages remains substantial. Of note, in sub-Saharan Africa, 10 countries had higher rates of estimated maternal mortality among women age 45 to 49 than in women aged 40–44, despite older women having lower fertility. Information on global abortion rates is not disaggregated by age [21].

**Table 3:** Percent of women aged 40–49 years from specified sub-Saharan African countries not using contraception with their fertility rates and estimated maternal mortality, the Demographic and Health Surveys (Source: http://dhsprogram.com/)

						Estimated maternal	
	Not using contraception			Fertility rates		Mortality per 1000 women	
Country	All ages	40–44	45–49	40–44	45–49	40–44	45–49
Sub-Saharan Africa							
Benin 2011–2012							
All	86.0	84.9	88.7	63	17		
Married	87.1	84.0	87.7				
Burkina Faso 2010							
All	84.7	82.6	90.1	87	23	0.7	0.7
Married	83.8	82.0	89.8				
Not married/Active	40.0	0.0	15.9				
Burundi 2010							
All	86.6	84.3	90.2	104	31	0.9	1.4
Married	78.1	80.5	86.7				
Cameroon 2011							
All	76.3	76.3	84.7	57	16	1.08	1.17
Married	76.6	74.8	82.1				
Not married/Active	41.6	75.0	73.5*				
Chad 2014–2015							
All	94.6	93.9	97.4	76	25	2.17	0.80
Married	94.3	94.0	96.9				
Comoros 2012							
All	86.3	82.1	91.4	63	28	0.04	0.00
Married	80.6	79.9	91.6				
Congo Republic 2011–2012							
All	55.7	60.8	81.6	61	9	0.3	0.8
Married	55.3	58.7	76.8				
Not married/Active	29.6	56.6	83.8				
Congo Democratic Republic 2013–2014							
All	80.7	79.6	88.5	97	20	2.55	0.82
Married	79.6	77.6	76.8				
Not married/Active	57.9	65.5	92.6*				
Cote d’Ivoire 2011–2012							
All	80.3	80.7	90.6	77	24	1.19	1.36
Married	81.8	80.1	89.3				
Equatorial Guinea 2011							
All	86.4	87.8	89.3	67	10	0.62	0.97
Married	87.4	87.9	91.0				
Not married/Active	80.7	85.2*	82.5*				
Ethiopia 2011							
All	80.4	79.7	89.1	68	28	1.43	0.70
Married	71.4	76.1	86.9				
Gabon 2012							
All	66.4	70.5	81.0	56	8	0.36	0.16
Married	68.9	74.0	79.6				
Not married/Active	43.8	33.5	87.6*				
Gambia 2013							
All	92.9	89.2	93.0	99	24	0.29	1.33
Married	91.0	88.9	92.3				
Ghana 2014							
All	77.2	76.6	85.1	52	17		
Married	73.3	74.8	81.7				
Guinea 2012							
All	91.5	94.7	97.2	73	32	0.82	0.69
Married	94.4	94.7	97.1				
Kenya 2014							
All	57.4	51.6	62.7	38	9	0.50	0.22
Married	42.0	42.3	54.8				
Not married/Active	34.6	61.0*	35.4*				
Lesotho 2014							
All	51.1	45.8	64.8	49	4	0.15	1.31
Married	39.8	40.5	60.1				
Not married/Active	26.6	36.3*	52.1*				
Liberia 2013							
All	78.3	82.8	92.7	50	14	3.12	1.77
Married	79.8	83.2	92.4				
Not married/Active	63.1	73.3*	86.2*				
Madagascar 2008–2009							
All	68.3	62.1	79.4	63	13	0.7	0.5
Married	60.1	55.9	74.1				
Not married/Active	56.9	66.7*	87.3*				
Malawi 2010							
All	64.6	55.9	62.8	82	33	2.1	1.5
Married	53.9	49.6	56.6				
Mali 2012–2013							
All	90.0	88.9	94.0	84	44	0.72	0.40
Married	89.7	88.6	94.0				
Mozambique 2011							
All	87.7	91.0	94.0	84	36	0.46	0.85
Married	88.4	90.0	93.1				
Not married/Active	69.7	86.7	85.4				
Namibia 2013							
All	49.8	45.1	53.9	42	10	0.50	0.46
Married	43.9	42.5	47.4				
Not married/Active	22.2	22.0	25.9				
Niger 2012							
All	87.5	91.0	96.4	100	49	1.24	0.65
Married	86.1	90.3	96.1				
Nigeria 2013							
All	84.0	78.8	87.6	78	29	1.10	0.59
Married	84.9	78.3	86.8				
Not married/Active	31.9	41.4	53.6*				
Rwanda 2014–2015							
All	69.1	55.9	72.2	65	12	0.59	0.00
Married	46.8	43.1	58.4				
Sao Tome and Principe 2008–2009							
All	69.3	66.7	82.6	87	8	0.70	0.29
Married	61.6	62.3	78.7				
Senegal 2015							
All	83.1	73.2	82.2	83	16		
Married	76.7	71.4	81.4				
Sierra Leone 2013							
All	77.9	81.5	87.9	64	32	1.13	0.72
Married	83.4	81.8	87.2				
Not married/Active	40.8	63.0	66.0*				
Swaziland 2006–2007							
All	62.1	61.8	73.2	30	4	1.42	0.00
Married	49.4	57.7	65.6				
Tanzania 2010							
All	71.2	63.3	76.3	72	22	0.1	0.0
Married	65.6	60.3	72.7				
Togo 2013–2014							
All	80.7	78.9	88.1	68	29	0.80	0.13
Married	80.1	76.9	86.7				
Uganda 2011							
All	76.4	68.0	82.5	74	26	1.06	1.11
Married	70.0	62.5	79.5				
Zambai 2013–2014							
All	64.9	55.8	74.0	71	14	0.92	1.18
Married	51.0	48.0	67.1				
Zimbabwe 2010–2011							
All	58.7	53.7	67.0	35	12	1.1	0.8
Married	41.5	40.5	55.8				

*Figures are based on small numbers (25-49 unweighted cases)

## Commentary and recommendations

The median age of menopause is approximately 51 years of age with about 95% of women reaching their final menses by age 54 years [8, 22]. Limited national surveillance data are available in high-income countries about contraceptive practices of midlife women, while unmet need for contraception increases among midlife women in low and middle-income countries, despite women’s potential fertility until the time of menopause.

Examination of available data from large national studies in the US reveals that even in the presence of data for women over 40 years of age, these data are often incomplete with respect to the types of contraception women use or whether women have experienced hysterectomy or ovariectomy. The NHANES collects data on contraception from women up to 54 years of age, but does not inquire about use of sterilization, IUD/IUS, barrier or natural family planning methods. Until the current round of data collection, the NSFG data only extended to 44 year olds, providing no information about women aged 45–54 who are still potentially at risk of pregnancy. BRFSS data on family planning are limited to women younger than 45, although one state includes data on women up to age 50 years, providing no data on those 51–54 years of age.

Based on examination of these studies, we recommend that all studies of contraception using national or state population samples increase the upper age boundary to include women up to and including 54 years of age. In addition, we recommend that these studies include data on sterilization (both male and female), IUD/IUS use, contraceptive implants, barrier, and natural family planning methods as well as whether women are menopausal and sexually active. Recognition that some women employ multiple methods supports reporting commonly used combinations. Given that many midlife women have had experience with using contraceptive methods throughout their reproductive lifespan, we can learn from their contraceptive practice patterns about the types of contraceptives and sequences and combinations they use and prefer in late reproductive life and during the menopausal transition.

Global patterns of contraceptive use by midlife women differ across the US, Europe and Canada and across low and middle-income countries. Data from Europe indicate that women over 40 years most commonly rely on sterilization or LARC, but 1/3 use no contraceptive methods. The UK data indicate that over 70% of women 40–49 years of age use contraception with nearly 50% relying on sterilization. Barrier methods (especially condoms) account for about 20% of women’s use and IUD, oral contraceptive and natural family planning each about 10%. In contrast to the UK, in Canada only 33% of women always used contraception with 40% relying on condoms and 36% on sterilization. For women in low income countries unmet need for contraception remains high overall, but notably increases after age 40 years when rates of maternal mortality also increase. Given the increased risk of maternal mortality for women after age 40 years, further research is warranted about unmet need for contraception in this age group in low and middle income countries. In addition, evidence for US women underscores the increased risk of maternal mortality for midlife African American women compared to White women, indicating potential health disparities in contraceptive access. Data on the intendedness of births and barriers to contraceptive utilization during this life-stage is lacking.

Midlife women begin to experience an increasing prevalence of chronic illnesses such as hypertension and diabetes and thus risks associated with specific contraceptive choices also change. For midlife women who desire to prevent pregnancy, or to become pregnant [23], contraceptive counseling should incorporate assessment of risks and use of the WHO Medical Eligibility Criteria (MEC) for Contraceptive Use (http://www.who.int/reproductivehealth/publications/family_planning/MEC-5/en/) or the US MEC guidelines (https://www.cdc.gov/reproductivehealth/contraception/mmwr/mec/summary.html). However, given the lack of data, it is not possible to evaluate the public health risks associated with lack of adherence to these guidelines in at-risk midlife women.

Taken together, both the limited availability of data about midlife women’s contraceptive practices and the different patterns reported from around the globe provoke many questions for further research. With respect to the lack of data, one might inquire about the assumptions that are responsible for this failure to include all midlife women in national surveys or to enquire about all types of contraception in cohort studies. Do investigators assume that midlife women are not sexually active?, that they are unable or unlikely to become pregnant?, or that midlife women are solely concerned about the amelioration of menopausal symptoms? Is it the case that the focus of studies such as the NSFG has been on family growth, with contraceptive use of interest only because of its impact on fertility during the most fertile years?

Few pregnancies occur after age 45, thus it might seem inefficient to sample women aged 46–55. Nonetheless, there has been limited interest in defining in detail, changes in fertility, reproductive aging, and pregnancy experiences in women’s late reproductive years, or in evaluating contraceptive needs/issues of women in the waning years of fertility. The net result has been limited data on midlife women’s health. Another possible explanation is that epidemiologic cohort studies have focused solely on understanding midlife women’s risk for cancer and chronic disease and not considered women’s fertility, sexuality or contraceptive needs during this phase of the reproductive lifespan. A bias may exist about “too few women” being affected by issues related to contraception, hence a lack of interest in the contraception-related health concerns of women at midlife. Whatever the cause for the limited attention to contraceptive practices of women in the midlife, there is reason to redouble research efforts given the fact of continuing fertility of midlife women, decisions by some midlife women to become pregnant during their 40s and 50s alongside the continuing problem of unintended pregnancy, the continuing need for elective abortions and the increased risks of maternal mortality at this life-stage.

## Conclusion

Although women remain at risk of pregnancy until they reach menopause, often until their mid-50s, information about the prevalence of contraceptive use and women’s contraceptive preferences is lacking. While pregnancy rates decline substantially in the midlife, pregnancy complications and risk of maternal death increase. National data on contraceptive prevalence are needed from women at least through age 54 years in both high and low income countries, as well as data on availability of reproductive services competent to address the health concerns of women at this life-stage. In low income countries as well as in the US, developing reproductive health services for women during the late reproductive years may be an important corollary to reducing the continuing burden of maternal mortality.
